# Supplementary material for: Gravity-awareness: a deep learning and LLM framework for predicting human adaptation to altered gravitational environments
Source: Front Psychol. 2026 Sep 24;17:1735872. doi: 10.3389/fpsyg.2026.1735872 (PMC13646142; doi:10.3389/fpsyg.2026.1735872)
Supplement: Supplementary file 1 [file Data_Sheet_1.pdf]

Supplementary Material: EEG & Physio Dataset Creation Protocol

Bakytzhan Alibekov   Alina Gutoreva   Elisa Raffaella-Ferre

October 2025

1 Introduction

2 Methods

2.1 Synthetic gravity profile

A continuous scalar gravity trajectory $g(t) \in \mathbb{R}_{\geq 0}$ was generated to emulate parabolic-flight phases (baseline $\approx 1g$, reduced-g, and hyper-g) as a bounded, piecewise-smooth signal. Let $\{(\tau_j, \tau_{j+1})\}_{j=1}^{J}$ be contiguous phase intervals and assume $g(t)$ is C^1 within each interval, with short C^0 transitions at phase boundaries to limit spectral splatter. For each analysis window (defined below), store the time-average

$$\bar{g}_i \;=\; \frac{1}{T_w} \int_{t_i}^{t_i+T_w} g(t)\, dt.$$

2.2 Channels, sampling, and base EEG generator

Signals were synthesized on a standard 10–20 montage with channel set $\mathcal{K}$, $|\mathcal{K}| = N_{\text{ch}}$, at sampling rate f_s. For channel $k \in \mathcal{K}$, the base (non-μ) EEG component was modeled as colored noise with $1/f^{\beta}$ spectrum plus weak broadband cortical activity:

$$x_k^{\text{base}}(t) \;=\; (h_\beta * \eta_k)(t), \qquad \mathcal{F}\{h_\beta\}(\omega) \propto |\omega|^{-\beta/2}, \quad \eta_k \sim \mathcal{N}(0,1).$$

Independent white sensor noise was added post hoc: $\epsilon_k(t) \sim \mathcal{N}(0, \sigma_\epsilon^2)$.

2.3 μ-rhythm synthesis with gravity-dependent envelope

A narrowband μ component ($\sim$8–13 Hz) was injected from the outset, spatially concentrated over the sensorimotor strip. A stochastic carrier $u(t)$ was produced by band-pass filtering white noise around a center f_μ with bandwidth Δf_μ:

$$u(t) \;=\; (h_\mu * \xi)(t), \qquad \mathcal{F}\{h_\mu\}(\omega) = \begin{cases} 1, & |\omega - 2\pi f_\mu| \leq \pi \Delta f_\mu, \\ 0, & \text{otherwise}, \end{cases} \qquad \xi \sim \mathcal{N}(0,1).$$

Topography was imposed via nonnegative weights w_k peaked at C3/Cz/C4 and decaying with geodesic distance. Gravity modulated the instantaneous μ amplitude through a deterministic envelope $A_\mu(g)$ defined by piecewise-linear interpolation over anchor pairs $\{(g_m, a_m)\}_{m=1}^{M}$:

$$A_\mu(g) \;=\; \text{interp1}\left(\{g_m\}, \{a_m\};\, g\right), \qquad a_m \geq 0.$$

The μ contribution at channel k was

$$x_k^{\mu}(t) \;=\; w_k\, A_\mu(g(t))\, u(t).$$

This yields sensorimotor μ ERD/ERS as a function of loading, consistent with movement-related rhythm principles.

2.4 Complete per-channel signal

$$x_k(t) \;=\; x_k^{\text{base}}(t) \;+\; x_k^{\mu}(t) \;+\; \epsilon_k(t).$$

2.5 Event timeline

Each phase interval (τ_j, τ_{j+1}) was annotated with a categorical label $c_j \in \{\text{baseline}, \text{reduced-}g, \text{hyper-}g\}$. For any t, the active label is $c(t) = c_j$ if $t \in (\tau_j, \tau_{j+1})$. Labels are stored alongside per-window summaries.

2.6 Preprocessing

A zero-phase band-pass filter H_{bp} was applied to each channel:

$$x_k^{\text{bp}}(t) \;=\; (H_{\text{bp}} * x_k)(t), \qquad H_{\text{bp}}:\; 1\text{–}45\ \text{Hz}.$$

When required by mains frequency, a narrow notch at 50 Hz (or 60 Hz) was applied.

2.7 Sliding windows

Signals were segmented into overlapping windows of duration T_w seconds with stride T_s seconds. For window index i with support $[t_i, t_i + T_w)$ and channel k, the windowed data are

$$x_k^{(i)}[n] \;=\; x_k^{\text{bp}}(t_i + n/f_s), \qquad n = 0, \ldots, N_w - 1, \quad N_w = T_w f_s.$$

2.8 Spectral estimation and band-power features

Per window i and channel k, the power spectral density (PSD) was estimated with Welch's method using a Hann segment of length L samples and overlap $p \in [0, 1)$. Denote $\widehat{S}_{k,i}(f)$ the PSD estimate. Band-limited powers were computed by integrating over canonical ranges:

$$P_{k,i}^{\alpha} = \int_8^{13} \widehat{S}_{k,i}(f)\, df, \qquad P_{k,i}^{\mu} = \int_8^{13} \widehat{S}_{k,i}(f)\, df,$$
$$P_{k,i}^{\beta} = \int_{13}^{30} \widehat{S}_{k,i}(f)\, df, \qquad P_{k,i}^{\gamma} = \int_{30}^{45} \widehat{S}_{k,i}(f)\, df.$$

Region-of-interest (ROI) summaries used convex weights v_k^{ROI} (e.g., sensorimotor mask for μ, frontal for β):

$$P_{\text{ROI},i}^{b} \;=\; \sum_{k \in \mathcal{K}} v_k^{\text{ROI}}\, P_{k,i}^{b}, \qquad b \in \{\alpha, \mu, \beta, \gamma\}, \quad \sum_k v_k^{\text{ROI}} = 1.$$

2.9 Subject-wise baseline and relative change

For each subject s, ROI, and band b, a baseline power $P_{\text{ROI},0}^{b}(s)$ was computed by averaging windows with label $c(t) = \text{baseline}$ and $\bar{g}_i$ within a small tolerance of $1g$. Relative (%) effects per window were

$$Y_{\text{ROI},i}^{b}(s) \;=\; 100 \cdot \frac{P_{\text{ROI},i}^{b}(s) - P_{\text{ROI},0}^{b}(s)}{P_{\text{ROI},0}^{b}(s)}.$$

This normalization removes static inter-subject scale and emphasizes gravity-linked ERD/ERS dynamics.

2.10 Stored records

For each window i, the dataset stores

$$(\bar{g}_i,\ c_i,\ Y^{\alpha}_{\mathrm{DMN},i},\ Y^{\mu}_{\mathrm{SMN},i},\ Y^{\beta}_{\mathrm{PFC},i},\ Y^{\gamma}_{\mathrm{global},i}),$$

along with identifiers (subject, run, time index). PSD hyperparameters (L, p), filter specifications, μ synthesis parameters $(f_\mu, \Delta f_\mu, \{(g_m, a_m)\}, \{w_k\})$ and ROI masks are saved as metadata to ensure deterministic regeneration and reproducibility. The μ component is present from the beginning of signal generation by construction of $x^{\mu}_k(t)$, ensuring that downstream band-power statistics reflect envelope–gravity coupling rather than offline augmentation.

2.11 Notes on downstream modeling features

No learning or loss functions are part of the dataset-creation pipeline. Gravity summaries $\bar{g}_i$ are recorded as scalar covariates only; any Fourier-feature encodings of g used by subsequent models are outside this section.

2.12 Gravity→Physiology formulas (pre-training)

Gravity anchors and admissible domain. A scalar gravity variable $g \in [0, 1.8]$ was restricted to the nominal parabolic-flight anchors

$$\mathcal{G} = \{\, 0,\ 0.16,\ 0.38,\ 1.0,\ 1.6,\ 1.8 \,\}.$$

All observations in v3 were mapped to elements of $\mathcal{G}$. Phase identity (e.g., hyper-g pull-up vs. pull-out) was collapsed to the common anchor $g = 1.8$.

Anchor statistics per target. Let y denote any retained target (HRV, BPV, EDA, or Activity). For each $g \in \mathcal{G}$ with available data, define the anchor mean

$$a_y(g) \ :=\ \mathrm{mean}\{\, y_i \ :\ g_i = g \,\}.$$

The available anchors for target y are

$$\mathcal{G}_y \ =\ \{\, g \in \mathcal{G} \mid a_y(g) \text{ is defined} \,\} \ =\ \{g_1 < g_2 < \cdots < g_{M_y}\}.$$

Piecewise interpolant (deterministic). For any $g \in [0, 1.8]$, define the descriptive gravity→feature curve as the clamped, shape-preserving interpolant through $\{(g_m, a_y(g_m))\}_{m=1}^{M_y}$:

$$f_y(g) \ =\ \begin{cases} a_y(g_1), & g \le g_1, \\ \mathrm{MC}^{\mathrm{Hermite}}(\{(g_m, a_y(g_m))\}_{m=1}^{M_y};\, g), & g \in [g_1, g_{M_y}], \\ a_y(g_{M_y}), & g \ge g_{M_y}, \end{cases}$$

where $\mathrm{MC}^{\mathrm{Hermite}}$ denotes the monotone cubic Hermite interpolant (Fritsch–Carlson construction). When $M_y < 3$, this reduces to linear interpolation between available anchors.

Percent-change view (for figures). For presentation, curves may be expressed as percent change relative to $1g$:

$$\Delta_y(g)\ [\%] \ =\ 100\Big(\frac{f_y(g)}{f_y(1.0)} - 1\Big),$$

provided $f_y(1.0)$ is defined.

Coupled identities (reporting only). For completeness, the physiological identity linking mean NN interval and heart rate is

$$\mathrm{HR}\,[\mathrm{bpm}] \;\approx\; \frac{60{,}000}{\mathrm{meanNN}\,[\mathrm{ms}]},$$

but in the descriptive construction, f_{meanNN} and f_{HR} are derived independently from their respective anchor means.

Targets covered. Apply $f_y(g)$ identically to each v3 target:

HRV: $y \in \{\mathrm{SDNN}\,[\mathrm{ms}],\ \mathrm{RMSSD}\,[\mathrm{ms}],\ \mathrm{meanNN}\,[\mathrm{ms}],\ \mathrm{HR}\,[\mathrm{bpm}]\}$.
BP/BPV (SBP): $y \in \{\mathrm{SBP}_{\mathrm{mean}}\,[\mathrm{mmHg}],\ \mathrm{SBP}_{\mathrm{HF}}\,[\mathrm{mmHg}^2]\}$.
EDA/GSR: $y \in \{\mathrm{SCL}_{\mathrm{R}}\,[\mu\mathrm{S}],\ \mathrm{SCL}_{\mathrm{L}}\,[\mu\mathrm{S}],\ \Delta\mathrm{SCL}_{\mathrm{R-L}}\,[\mu\mathrm{S}]\}$.
Activity: $y \in \{\mathrm{TrunkAct}\,[\mathrm{counts/s}],\ \mathrm{WristAct}\,[\mathrm{counts/s}]\}$.

Handling of missing anchors. If a target lacks values at some $g \in \mathcal{G}$, use $\mathcal{G}_y$ as-is. The interpolant spans $[g_1, g_{M_y}]$ only; values outside are clamped to the nearest available anchor mean as specified above.

Purpose and scope. The formulas $f_y(g)$ are descriptive (non-learned): they summarize gravity trends embedded in v3 for shape checks, figure generation, and optional normalized views $\Delta_y(g)$. They do not incorporate subject effects, task differences, or model training.

References

- Pfurtscheller, G., & da Silva, F. L. (1999). Event-related EEG/MEG synchronization and desynchronization: Basic principles. *Clinical Neurophysiology*, 110(11), 1842–1857.
- Rahimi, A., & Recht, B. (2008). Weighted sums of random kitchen sinks: Replacing minimization with randomization in learning. *Advances in Neural Information Processing Systems*, 21.
- Tancik, M., Srinivasan, P., Mildenhall, B., Fridovich-Keil, S., Raghavan, N., Singhal, U., ... & Ng, R. (2020). Fourier features let networks learn high-frequency functions in low-dimensional domains. *NeurIPS*, 33, 7537–7547.
- Welch, P. D. (1967). The use of fast Fourier transform for the estimation of power spectra: A method based on time averaging over short, modified periodograms. *IEEE Transactions on Audio and Electroacoustics*, 15(2), 70–73.
- Fritsch, F. N., & Carlson, R. E. (1980). Monotone piecewise cubic interpolation. *SIAM Journal on Numerical Analysis*, 17(2), 238–246.
